# Predictors of physical activity following gestational diabetes: Application of health action process approach

**DOI:** 10.1002/nop2.486

**Published:** 2020-05-21

**Authors:** Banafsheh Mohammadi Zeidi, Nourossadat Kariman, Zahra Kashi, Isa Mohammadi Zeidi, Hamid Alavi Majd

**Affiliations:** ^1^ Student Research Committee Department of Midwifery and Reproductive Health School of Nursing and Midwifery Shahid Beheshti University of Medical Sciences Tehran Iran; ^2^ Midwifery and Reproductive Health Research Center Midwifery and Reproductive Health Department School of Nursing and Midwifery Shahid Beheshti University of Medical Sciences Tehran Iran; ^3^ Diabetes Research Center Imam Khomeini Hospital Mazandaran Iran; ^4^ Department of Public Health School of Health Qazvin University of Medical Sciences Qazvin Iran; ^5^ Department of Biostatistics Paramedical School Shahid Beheshti University of Medical Sciences Tehran Iran

**Keywords:** determinants, gestational diabetes, nurses, nursing, physical activity, postpartum

## Abstract

**Aim:**

Regular physical activity can reduce the chance of developing type 2 diabetes in women with a history of gestational diabetes. The present study investigated the relationship between the constructs of the health action process approach and regular physical activity in women with a history of gestational diabetes.

**Design:**

This was a cross‐sectional study.

**Methods:**

A total of 150 women who had given birth 6–24 months prior to the study and had experienced gestational diabetes in their recent pregnancy were selected using multistage cluster sampling. Data were collected from December 2018 to May 2019 using a researcher‐made questionnaire including constructs of health action process approach.

**Results:**

The common fit indices revealed that health action process approach had an acceptable fit to the observations (root mean square error of approximation = 0.054, Tucker–Lewis index = 0.95, comparative fit index = 0.955). The model's constructs predicted 48% of intention variance and 35% of physical activity variance. Action self‐efficacy and coping planning were the most important predictors of intention and behaviour, respectively.

## 1 INTRODUCTION

Type 2 diabetes is a health problem in many countries, especially developing ones (Guo, Tang, Wiley, Whittemore, & Chen, 2018). The International Diabetes Federation (IDF) estimates that at least 425 million people worldwide have diabetes. From 1980–2014, the incidence of diabetes more than doubled in males and increased by almost 60% in females. If this trend continues, the WHO goal to stop the increase in diabetes by 2025 will not be achieved. Diabetes can be prevented or delayed by changing the lifestyle of high‐risk individuals (Goveia et al., 2018). Since the risk of diabetes in women with a history of gestational diabetes is seven times that of women who had normal blood sugar levels during pregnancy (Buelo, Kirk, Lindsay, & Jepson, 2019), gestational diabetes provides a unique opportunity to prevent type 2 diabetes (Goveia et al., 2018).

Studies have shown that physical activity improves glucose homeostasis in various ways (Wang, Guelfi, & Yang, 2016).Therefore, women with gestational diabetes are advised to do moderate‐intensity physical activity for at least 30–60 min three times a week (Padayachee & Coombes, 2015). They are also advised to follow the same instructions after childbirth. Doing postpartum physical activity without adversely affecting milk volume and composition increases cardiovascular fitness, improves mood and accelerates reaching and maintaining the ideal weight. Having regular physical activity after childbirth, when combined with calorie restriction, can prevent or delay diabetes in women who have already had gestational diabetes (Di Biase et al., 2019). The results of a cohort study showed that the risk of developing type 2 diabetes was 47% lower in women who had previously had gestational diabetes and performed postpartum moderate‐intensity physical activity for 150 min a week (Buelo et al., 2019).

The optimal time to reform one's lifestyle is 6–24 months postpartum (Peacock et al., 2015). However, evidence suggests that only about one‐third of women with a history of gestational diabetes are physically active during this period (Graco, Garrard, & Jasper, 2009; Koh, Miller, Marshall, Brown, & McIntyre, 2010; Koning et al., 2016).

Healthcare professionals including nurses have important role in minimizing the risk for development of chronic disease. Unfortunately, opportunity for health promotion and prevention of diabetes is often missed by healthcare professionals and many women with gestational diabetes history receive little or no intervention that would minimize their risk of developing diabetes after giving birth (Aluş Tokat, Sancı, Girgeç, Kulhan, & Özcan, 2016).

Health education theories are powerful methodological tools for changing health behaviours including physical activity, because they can explain and predict a phenomenon and its effective factors. They are, therefore, widely used in health behaviour research (Thompson, Vamos, & Daley, 2017).

One of the models contributing to a better understanding of factors influencing behaviour change is the health action process approach (HAPA; Schwarzer, 2008). This model seeks to address deficiencies in models such as planned behaviour and social cognition theory, because these theories try to identify and modify intention predictors, while forming a strong intention does not necessarily lead to behaviour change (Barg et al., 2012). According to this theory, passing through two stages (motivational and voluntary) is essential for the formation of healthy behaviour in a person. In the first or motivational phase, risk perception, outcome expectancies and action self‐efficacy will lead to the formation of intention to adopt preventive behaviour or to change risky behaviour (MacPhail, Mullan, Sharpe, MacCann, & Todd, 2014). When behavioural intention is formed, the individual enters the voluntary phase. In the second or voluntary stage, factors such as action planning, coping planning, maintenance self‐efficacy and recovery self‐efficacy will lead to turning the intention to behaviour and to maintaining that behaviour (Schwarzer, 2008).

## 2 BACKGROUND

Recent studies in Iran have shown that the HAPA model can be used to predict students’ nutritional behaviour (Alinaghizadeh, Javadi, & Lesani, 2017), nutritional behaviour and physical activity in people with type 2 diabetes (Rohani, Bidkhori, Eslami, Sadeghi, & Sadeghi, 2018; Rohani, Sadeghi, Eslami, Raei, & Jafari‐Koshki, 2018), parenting skills in mothers (Norouzi et al., 2015) and breast self‐assessment (Ghofranipour, 2018). However, a theory should be tested to identify the factors that influence favourable behaviour change in the target group (Rohani et al., 2015).

### 2.1 Aim

This study aimed to identify predictors of physical activity in women with a history of gestational diabetes.

### 2.2 Design

This was a descriptive cross‐sectional study conducted from December 2018–May 2019 in Mazandaran Province in Iran.

## 3 METHODS

Considering the 5–10 samples required for each variable entering the model in studies using structural equations for data analysis and also considering the time and cost and the nine variables in the current study, the sample size of 150 individuals was determined. Participants were selected through multistage cluster sampling from among women who referred to urban and rural healthcare centres provided they had a history of diabetes in their recent pregnancy and were willing to participate in the study. For this purpose, Mazandaran Province was first divided into three regions. Then, two regions were randomly selected. Next, two districts were randomly selected from each region. According to the total sample size calculated and the prevalence of gestational diabetes in each district, the sample size was calculated for each of them. Then, women who met the inclusion criteria were selected randomly from urban and rural health centres in the selected districts. The exclusion criteria were having diabetes (type 1 or type 2), known mental illness, or cancer (according to participants), smoking and/or substance abuse and pregnancy or the intention to become pregnant within the next 6 months.

A self‐report researcher‐made questionnaire with two parts was used for data collection. The first part included demographic questions such as age, educational level, employment status, history of insulin use, during pregnancy and BMI; the second part included 30 items aimed at measuring HAPA constructs. Of the 30 items, two were related to risk perception, three to outcome expectancies, four to action self‐efficacy, three to intention, four to action planning, four to coping planning, seven to maintenance self‐efficacy and three to recovery self‐efficacy constructs. Most items were answered based on a 5‐point Likert scale from 1 = strongly disagree to 5 = strongly agree; however, the risk perception construct was scored based on a 5‐point Likert scale from 1 = very low to 5 = very high, and self‐efficacy constructs were scored based on a 5‐point Likert scale from 1 = not sure at all to 5 = completely sure. The International Physical Activity Questionnaire‐Short Form (IPAQ‐SF) was used to measure physical activity. Participants were asked to determine the frequency and duration of their physical activity over the prior 7 days at the three levels of intense (such as aerobic), moderate (such as moderate‐speed cycling) and walking by self‐reporting. Then, the total amount of physical activity was calculated in terms of MET‐minutes/week. The validity of IPAQ‐SF is verified by the accelerometer model portable monitor device and is validated in 12 countries (Craig et al., 2003). Psychometric evaluation of IPAQ‐SF in Iran was done by Baghbani Moghadam et al., and its internal consistency using Cronbach's alpha coefficient was .7, indicating good internal consistency. Its reliability over time through the test–retest method with a two‐week interval using Spearman–Brown correlation coefficient was .9, indicating good reliability (Moghaddam et al., 2012).

The questionnaires were provided to the participants after the research objectives were explained to them, informed consent was obtained, and information confidentiality was emphasized. When participants completed the questionnaires, a member of the research team was present on‐site to answer any questions. In addition, provision of accurate and complete answers to questions was ensured.

The content validity of the researcher‐made questionnaire was verified by quantitative and qualitative methods. In the qualitative method, the pilot form of the questionnaire was provided to 10 experts in reproductive health, health education, physical education and instrumentation and they were asked to rank the items and provide feedback based on the use of appropriate words, item placement, grammar compliance and appropriate scoring. The content validity ratio (CVR) and content validity index (CVI) were calculated for quantitative verification of content validity (Karimy, Niknami, Heidarnia, & Hajizadeh, 2012). CVR was determined by asking experts about the necessity of items, and values above 0.62 were accepted based on the Lawshe table. The relevance, clarity and simplicity of each item were evaluated to determine CVI, and values above 0.79 were accepted. The CVR of the questionnaire was 0.893, its CVI was 0.98, and six items were deleted due to CVR scores below 0.62. Face validity was also evaluated both qualitatively and quantitatively. In the qualitative method, the questionnaire was randomly distributed among 20 individuals in the target group who were not participating in the study. They were asked to study the questions carefully and determine the level of difficulty, irrelevance and any ambiguities (Ardestani, Niknami, Hidarnia, & Hajizadeh, 2017). Based on the comments and suggestions received from these individuals, changes were made to clarify the items. The impact score was used in the quantitative method to exclude inappropriate items and determine the importance of each item. An impact score above 1.5 was considered acceptable (Foroumandi, Alizadeh, Hajizadeh, Haghravan, & Mohajeri, 2018).

Internal consistency and stability were used to determine the reliability of the questionnaire. For this purpose, the questionnaire was distributed among 30 members of the target group and Cronbach's alpha value was calculated after collecting and extracting data to assess internal consistency. Values of .7 and above were considered satisfactory. The test–retest method with a two‐week interval was used to check the reliability of the questionnaire. Then, the consistency of the questionnaire was determined using intra‐cluster correlation coefficient (Foroumandi et al., 2018). In this study, Cronbach's alpha of the questionnaire was 0.82 (−0.76–0.84) and total ICC was 0.88 (0.75–0.96).

### 3.1 Analysis

SPSS‐23 software was used to calculate the frequency distribution, mean and standard deviation and correlation matrix of the main variables. Structural equation modelling using AMOS‐23 software was used to test the model fit and to investigate the relationships between variables in the model. The covariance matrix was performed with the maximum likelihood method, and full information maximum likelihood (FIML) was used to manage the missing data. The most important indices of the model adequacy assessment were the chi‐square to degrees of freedom (*χ* ^2^/*df*), root mean square error of approximation (RMSEA), comparative fit index (CFI), Tucker–Lewis index (TLI) and incremental fit index (IFI).

### 3.2 Ethics

The code of Research Ethics Committee approval for this study is IR.SBMU.RETECH.REC.1397.831.

## 4 RESULTS

The mean age of participants was 33.50 (*SD* 5.06) years. Almost half (44.7%) of them had a high school diploma, most (82.7%) were housewives, and most had a BMI > 25 (82.6). Other descriptive statistics are presented in Table 1.

**TABLE 1:** Socio‐demographic characteristics of research participants

Demographic variables	Per cent	Number	
Age (year)	30	45	20–30
	3.61	92	30–40
	8.7	13	≥40
Educational level	10.7	16	Primary school
	18	27	Secondary school
	1.3	2	High school
	44.7	67	High school diploma
	25.3	38	Academic education
Employment status	82.7	124	Housewife
	17.3	26	Employed
History of insulin use during pregnancy	64	96	Yes
	36	54	No
Body mass index (BMI)	17.3	26	<25
	39.3	59	25–30
	43.3	65	>30
Physical activity	57.3	86	No physical activity
	26	39	Low‐intensity physical activity
	16.7	25	Moderate‐ to high‐intensity physical activity

The mean and standard deviation of HAPA model constructs are presented in Table 2.

**TABLE 2:** The mean and standard deviation of HAPA model constructs

Construct (scoring range: 1–5)	No of items	Mean and *SD*
Risk perception	2	3.2 ± 1.21
Outcome expectancies	3	4.14 ± 0.8
Action self‐efficacy	4	2.93 ± 1.28
Intention	3	2.81 ± 1.17
Action planning	4	2.38 ± 1.47
Coping planning	4	1.88 ± 1.29
Maintenance self‐efficacy	7	2.46 ± 1.22
Recovery self‐efficacy	3	2.92 ± 1.51

**FIGURE 1:**
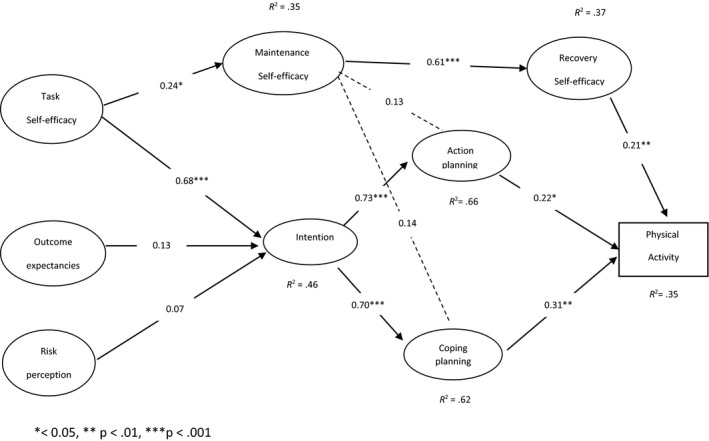
Structural model of physical activity predictors in women with history of gestational diabetes

The Pearson correlation coefficient between HAPA constructs showed significant correlations between all constructs except for risk perception (*p* < .01). In the motivational phase, the strongest significant correlation coefficient was observed between action self‐efficacy and intention (*r* = .558). In the voluntary phase, the strongest correlation coefficient was observed between coping planning and physical activity (*r* = .589). Table 3 shows the correlations between HAPA constructs. The results of structural equation modelling (Figure 1) showed that in general, the present study data had a good fit for the model used. Table 4 shows the model fit indices.

**TABLE 3:** Correlation between variables used in the HAPA model

HAPA constructs	1	2	3	4	5	6	7	8	9
1. Risk perception	1								
2. Outcome expectancies	.119	1							
3. Action self‐efficacy	.046	.164*	1						
4. Intention	.119	.268**	.587**	1					
5. Action planning	.087	.205*	.636**	.674**	1				
6. Coping planning	.090	.223*	.576**	.656**	.796**	1			
7. Maintenance self‐efficacy	−.085	.148	.476**	.499**	.518**	.515**	1		
8. Recovery self‐efficacy	−.007	.178*	.505**	.461**	.445**	.443**	.579**	1	
9. Physical activity	−.030	.090	.507**	.455**	.539**	.589**	.490**	.445**	1

* Correlation is significant at .05.

** Correlation is significant at .01.

**TABLE 4:** Model fit indices

*χ* ^2^/*df*	RMSEA	CFI	IFI	TLI
1.437	0.054	0.955	0.955	0.950

In this study, HAPA model constructs calculated 48% of intention variance, 66% of action planning variance, 62% of coping planning variance and 35% of physical activity variance. In the motivational phase, the action self‐efficacy construct was significantly associated with the intention to adopt a healthy diet (*β* = .68 and *p* = .000). The risk perception and outcome expectancies constructs were not significantly correlated with intention (*β* = .072, *p* = 698 and *β* = .13, *p* = .061, respectively). In the voluntary phase, action planning (*β* = .22 and *p* = .017), coping planning (*β* = .31 and *p* = .001) and recovery self‐efficacy (*β* = .21 and *p* = .004) constructs were directly and significantly correlated with physical activity. In this model, the relationship between maintenance self‐efficacy construct and action planning (*β* = .13 and *p* = .085) and coping planning (*β* = .144 and *p* = .068) was not significant.

## 5 DISCUSSION

This study aimed to investigate factors affecting the physical activity of women with a history of gestational diabetes using HAPA. In this study, the physical activity of only 16.7% of women was at the moderate‐to‐severe (favourable) level after gestational diabetes. The prevalence of health‐promoting physical activity after gestational diabetes was 31%, 37.2% and 48.8% in studies by Kim, McEwen, Kieffer, Herman, and Piette (2008), Koh et al. (2010) and Kieffer, Sinco, and Kim (2006), respectively. One reason for this difference can be culture, as women are generally less physically active after childbirth than before it in Iran. Ouji, Barati and Bashirian (2014) reported that postpartum physical activity was only favourable in 17% of women. Other reasons are a lack of access to facilities or a lack of support from family and friends. In addition, training provided during pregnancy and postpartum does not include physical activity‐enhancing training (Ouji et al., 2014). In any case, low levels of physical activity following gestational diabetes should be considered as a serious warning sign and necessary interventions should be designed to promote physical activity and ultimately prevent type 2 diabetes.

In line with most studies using HAPA, the strongest predictor of intention to perform physical activity in the present study was action self‐efficacy (Barg et al., 2012; Paxton, 2016; Pinidiyapathirage, Jayasuriya, Cheung, & Schwarzer, 2018). This finding implies that a woman's belief in her abilities is one of the most important factors in shaping intention before starting action. Therefore, action self‐efficacy needs to be promoted in different ways (such as sharing experiences of successful people, motivations and setting achievable goals) to improve intention (Chiu, Lynch, Chan, & Berven, 2011).

In this study, unlike the HAPA model, the risk perception construct had no significant relationship with intention, which could be due to the risk assessment tool, because the researchers evaluated only the risk of future type 2 diabetes and no other gestational diabetes risks, such as recurrence of diabetes in a subsequent pregnancy or cardiovascular diseases. Given similar findings in some studies (Barg et al., 2012; Crawford, Terry, Ciro, Sisson, & Dionne, 2018; Pinidiyapathirage et al., 2018; Rohani, Sadeghi, et al., 2018), especially in Zhang's meta‐analysis (Zhang, Zhang, Schwarzer, & Hagger, 2019), it may be concluded that the risk perception construct is of little use in changing preventive health behaviours alone or in the absence of other motivational factors (Renner et al., 2008). In contrast, it is more valuable in behaviours such as cancer screening or breast self‐assessment that lead to disease diagnosis (Schwarzer et al., 2007).

In this study, unlike the HAPA model, no significant relationship was found between outcome expectancies and intention. The absence of a significant relationship between the two constructs was reported in studies by Paxton (2016) and Pinidiyapathiage, Jayasuriya, Cheung and Schwarzer (2018). In other studies with similar objectives, however, the relationship between the two constructs was significant (Barg et al., 2012; Crawford et al., 2018; Harman, 2014). This inconsistency might be due to differences in demographic characteristics such as participants’ age, as the expected positive outcomes appear to be a strong predictor of physical activity in the elders, but not in young and middle‐aged individuals (Williams, Anderson, & Winett, 2005).

Consistent with the HAPA model, intention had a significant relationship with action planning and coping planning in the current study. This finding, along with the significant relationship between the two planning constructs with physical activity, implies the mediating role of planning. In other words, without the planning construct, intention will not become behaviour. Action planning and coping planning are self‐regulatory strategies that play a vital role in adopting and maintaining healthy behaviours. In the present study, physical activity had a stronger construct relationship with coping planning than with action planning. In other words, the strongest predictor of physical activity in the target community was coping planning.

In the present study, the HAPA model constructs explained 35% of the variance in behavioural changes, indicating the effectiveness of the HAPA model in predicting factors affecting physical activity in the target community. This figure was approximately like that in studies by Paxton (2016), Caudroit, Stephan, and Scanff (2011) and Crawford et al. (2018) and was higher than that in studies by Pinidiyapathirage et al. (2018) and Barg et al. (2012). The coefficient of determination was 42%, 39%, 28%, 15% and 11% in studies by Paxton (2016), Caudroit et al. (2011), Crawford et al. (2018), Pinidiyapathirage et al. (2018) and Barg et al. (2012), respectively, and was the strengths of the present study. This study is also the first in Iran to use the HAPA model to predict factors affecting physical activity in women with a history of gestational diabetes.

### 5.1 Limitations

This study had some limitations. In this study, physical activity was assessed as self‐reported, which may not reflect one's physical activity as it is. This was a cross‐sectional study. In this type of study, causal relationships cannot be investigated, and only simple relationships can be assessed. Therefore, it is recommended that further studies be designed and implemented in a prospective or interventional way.

## 6 CONCLUSION

The results of this study, along with those of other studies, provide evidence that HAPA is useful in predicting preventive behaviours.

## CONFLICT OF INTEREST

There are no conflicts of interest associated with this study.
