# Corrigendum

**DOI:** 10.1002/nop2.595

**Published:** 2020-08-03

In Mohammadi Zeidi et al. (2020), the affiliation for Zahra Kashi is incorrect. The author's affiliation should be amended from ^3^Diabetes Research Center, Imam Khomeini Hospital, Mazandaran, Iran, to ^3^Diabetes Research Center, Mazandaran University of Medical Sciences, Sari, Iran.

The authors apologize for this error.
